# The Education of Playful Boys: Class Clowns in the Classroom

**DOI:** 10.3389/fpsyg.2018.00232

**Published:** 2018-03-01

**Authors:** Lynn A. Barnett

**Affiliations:** Recreation, Sport and Tourism, University of Illinois at Urbana–Champaign, Champaign, IL, United States

**Keywords:** children's playfulness, class clown, disruptiveness, classroom behavior, teacher-student relationship

## Abstract

This longitudinal study identified degrees of playfulness in 278 kindergarten-aged children, and followed them through their next three school years to determine how playfulness was viewed by the children themselves, their classmates, and teachers. Perceptions of the social competence, disruptiveness, and labeling as the class clown, were assessed from all perspectives in each of first through third grades. Hierarchical linear modeling was conducted to account for the nesting of the data (children within classrooms within schools) and for the lack of independence between the measures. A central finding confirmed extant literature in that gender differences were dominant, with playful boys regarded as distinct from their less playful counterparts, while no such discrepancies appeared for girls. Playful boys were increasingly negatively regarded as rebellious and intrusive and were labeled as the “class clown” by their teachers. These findings were in direct contrast with children's self-perceptions and those of their peers, who initially regarded more playful boys as appealing and engaging playmates. The data further revealed that the playful boys were stigmatized by their teachers, and this was communicated through verbal and non-verbal reprimands, and classmates assimilated this message and became increasingly denigrating of the playful quality in the boys. In stark contrast, girls' playfulness levels were not a consideration in ratings by teachers or peers at any grade, nor did their classroom behaviors show significant variation. These negative perceptions were likely transferred by teachers to peers and to the children themselves, whereupon they changed their positive perceptions to be increasingly negative by third grade. The results contribute to the literature by demonstrating that playfulness in boys (but not girls) is often associated with the “class clown” designation, and is viewed as an increasingly lethal characteristic in school classrooms, where compelling efforts are undertaken to discourage its expression and persistence.

## Introduction

A stream of research has systematically investigated young children's playfulness by endeavoring to determine its underlying structure, dynamics, correlates, and nomological network (Lieberman, 1977; Barnett, 1990, 1991a,b). The most consistent findings have determined that there are five constituent determinants of the playfulness quality, and that their combined effect is highly predictive (Lieberman, 1966; Barnett, 1990, 1991a). The physical spontaneity dimension reflects the child's activity level and physical coordination; social spontaneity captures his or her ability to move in and out of social play situations fluidly, to share, and to show leadership during peer play; cognitive spontaneity reflects the degree to which imagination and creativity are shown in play by the child inventing games, roles, and characters; manifest joy is demonstrated by the degree of exuberance, joy, enthusiasm, and heightened positive emotions the child exhibits in play; and sense of humor encompasses the teasing, rhyming, humor appreciation, and joke-telling aspects shown during play.

Empirical studies have also sought to identify descriptive lexicons that appear to distinguish between children who have more and less of the playful quality (Singer et al., 1980). Barnett (1991b) found that the characteristics that differentiated high and low playful children were “bright,” “active,” “aggressive,” “curious,” “imaginative,” “impulsive,” “mischievous,” “cheerful,” “confident,” “dependent,” and “responsible.” These results were informed by children's teachers and recreation leaders, as well as by adult observers naive to the children they rated—so they appear to be communally understood. Rogers et al. (1998) obtained significant correlations with the personality descriptors of approachable, adaptable, persistent, aggressive, impatient, competitive, and dependent with similar aged children. Several of these characteristics appear to be at variance with the consistently positive ones that the public seems to identify.

### The advent of a structured setting: the formal school years

All of these empirical studies on children's playfulness have been conducted with very young children, spanning the ages of 2 to 6 years, and there has been a dearth of explorations into playfulness with school-aged children. The preschool and kindergarten settings contrast sharply with those comprising primary school grades in that they are much more relaxed and informal, and the degree of structure, the number of rules, and the degree of adult supervision and scrutiny are all less. It is therefore quite reasonable that virtually all of the extant research on children's playfulness has been conducted in permissive settings where manifest playful behaviors are plenteous.

The transition from kindergarten represents a process of significant and extensive adaptation in which children must quickly learn to follow directions, pay persistent attention, and internalize new expectations and rules (McClelland et al., 2007; Suchodoletz et al., 2009). Hence, the move to formal schooling requires that children transition to a more structured environment that demands self- discipline and -control. Children's ability to regulate their behavior is critically important, in that it has been shown to be predictive of how well they adapt to school (Blair, 2002), and to their academic achievement through the primary grades and middle school (McClelland et al., 2000, 2006, 2007; Vitaro et al., 2005). Research has demonstrated that how well children are able to navigate this transition also forecasts their long-term educational trajectory (McClelland et al., 2006). Conversely, children who experience substantial difficulty are at greater risk for poor academic achievement, problems with social peer relationships, emotional and conduct problems, and dropping out of school before or during adolescence (Eisenberg et al., 2000; McClelland et al., 2000; Vitaro et al., 2005).

Children's adjustment to the formal classroom setting is typically assessed by teachers using three metrics (Perry and Weinstein, 1998): academic functioning, social functioning, and classroom behavioral functioning, with the latter regarded as the most essential to school readiness (Petriwskyj et al., 2005). Successful adjustment is defined by teachers as accommodation to the classroom culture, rules, and behavioral expectations (Petriwskyj et al., 2005). To transition and function effectively requires that children be able to exercise self-control over their behaviors (McClelland et al., 2007) and restraint in expressing emotions (Diener and Kim, 2004). Teachers view children who they perceive to be distracting or disruptive as a detriment to the classroom learning environment, and they endeavor to control, shape or extinguish these behaviors in multiple ways (Jones and Dindia, 2004).

The characteristics that depict young playful children and expound on their exuberant, physically active, spontaneous, and impulsive qualities appear to be incompatible with the more restrictive school setting where rules and structure prevail and where the requirements for children to constrain their behaviors are intensified. The representations of playful kindergarteners would posit they might have a problem in negotiating a less familiar and more stringently controlled environment and one which demands well-developed behavioral self-regulation. This would portend that playful children might encounter problems successfully adapting to the classroom setting and maintaining obedience to classroom rules and teachers' demands. Playful children might well find themselves in conflict with their teacher, who—because of the emphasis on “appropriate” classroom behaviors—might view playful characteristics as disruptive and troublesome. It was thus a major focus of the present study to investigate how playful children transition to the primary school setting by investigating how they are perceived by their teachers, particularly the extent to which they are viewed as disruptive to classroom decorum. We also wondered whether—as the degree of structure present in the school classroom increased—the difficulties children would incur in trying to manage their behavior (stifle their playful expression) would also increase. We speculated that more playful children might be perceived as increasingly disruptive by their teachers as they progress through the first three primary school years.

#### Playful children and their classmates

The school classroom can also be regarded as a principal setting in which children interact with their peers (Rubin et al., 2006). Their ability to manage their behavior appropriately relates to their social competence, interpersonal skills, social status, and success in peer interactions (Vitaro et al., 2005; Trentacosta and Izard, 2007). Conversely, children's difficulties normalizing their behavior have been shown to cause problems with forming friendships, and more generally in developing social competence (McClelland et al., 2000).

Research has also shown that teachers exert influence on children's peer relationships (Hughes et al., 2001) by providing social cues about how likeable a peer is (Hughes et al., 2001; Farmer et al., 2011). The teacher's prominent visible role in the classroom provides extensive opportunities for students to observe exchanges with classmates, and to develop ideas about those who are liked and disliked, which then influences their own affective evaluations (Hughes et al., 2001, 2014). In accordance with social referencing theory, Hughes et al. (2001, 2014) found that students reported liking classmates who they viewed as having a positive relationship with the teacher, and disliking those they observed to be conflictual. Their findings emphasize the significant role the teacher plays in serving as a socializing agent who can significantly influence children's social relationships with peers.

It is thus crucial to children's social development and status that they are perceived by their teacher as agreeable and affable. Classmates will recognize children whose relations and exchanges with their teacher are disapproving, and they may adjust their impressions to dislike or shun these peers. The children who are most likely to have negative interactions with their teachers are those with a higher tendency to show disruptive classroom behavior and less able to constrain or redirect their frequent offtask activities (Kean, 1995; Rothbart and Bates, 2006). Their teachers come to regard them as less competent academically, and more challenging to manage and teach (Rothbart and Bates, 2006). The characteristics that have been identified as distinguishing playful children, including the propensity to be more physically active (Lieberman, 1977; Singer et al., 1980; Barnett, 1991b), verbal (Singer et al., 1980), impulsive (Barnett, 1991b; Rogers et al., 1998), aggressive (Barnett, 1991b; Rogers et al., 1998), and mischievous (Barnett, 1991b), would portend a poor relationship with teachers, which could then be transmitted to their classmates. Consistent with this literature, we wondered if more playful children would, at least initially, be viewed by their peers different in social status than children who are less playful, and whether their views would change across time (grades).

Alternatively, in studies with middle school children, research has demonstrated that there is often a difference in perspective between children and their teachers. One such area in which divergences have been detected is in the extent to which various classroom behaviors are viewed as disruptive, and how serious misbehaviors are judged to be. While some studies have found teachers to regard disobediences as more unforgiving (Corsaro and Eder, 1990), other research has shown students attach harsher views of classroom transgressions (Dursley and Betts, 2015). What is significant for the present study is the divergence between the perceptions of students and their teachers, particularly in evaluations of disruptive behaviors in the classroom—one central focus of this study. We thus sought separate assessments regarding the extent to which incidences of classroom behavior might be considered disruptive, to consider the perspectives of teachers and students individually, and to examine the role of children's playfulness as predictive of any differences.

#### How playful children view themselves

After little more than a month in their classroom, children as young as first graders have been shown to make inferences about their own abilities from cues provided within the classroom (Stipek, 1981; Stipek and Tannatt, 1984). They attend to the differential ways in which teachers respond to other students, how and where praise and criticism are overtly rendered, responses to questions that are asked, how assessments and grades are assigned, and groupings of students based on ability (Jussim, 1986; Weinstein et al., 1987). They are keenly aware of the academic and social expectations of their teacher and the differential treatment that ensues from varying degrees of compliance, and they ultimately adopt these expectations as their own (Weinstein et al., 1987).

Numerous studies have demonstrated that teachers may formulate expectations for their students based on their classroom behaviors (Dusek and Joseph, 1983), and that these expectations can influence students' performance and motivation (for a review see Jussim and Harber, 2005). Known as the “Pygmalion Effect” (Rosenthal and Jacobson, 1968) in educational research, it has been shown that teachers may form initial expectations for a student, and they then behave toward the student in accord with their expectations. In turn, students respond to the ways in which they are treated by teachers, and ultimately they may internalize these expectations, with the result that a self-fulfilling prophecy is evinced. While an initial teacher-held expectation may be erroneous in whole or in part, through numerous interactions students will come to behave in such a manner as to confirm the expectation, which then becomes more accurate. In the present context, we hypothesized that teachers who view playful students as a problem in the classroom may hold differential behavioral expectations consistent with their perception, and students may come to adopt these negative behaviors, and regard themselves in corresponding ways (Weinstein, 2002; Jussim et al., 2009; McKown et al., 2010). We thus endeavored to explore the extent to which more playful students were affected by the assessments held by their teachers, and how readily this might have transpired. We included students' self-assessments of the social and behavioral constructs evaluated by teachers in each grade to determine whether any transmittals occurred, and how quickly and to what extent. In concert with the Pygmalion effect and a self-fulfilling prophecy to which it may lead, we inquired as to whether playful children would come to perceive themselves to be disruptive in the classroom, consonant with the perceptions of their teachers.

#### The clown in the classroom

There are typically students in every classroom who are considered disruptive because they use humor in the form of jokes, gestures, and antics with the goal of amusing or entertaining other students, and they have been often been labeled “class clown.” In comparisons to non-clowns, they have the common signature strength of generating and appreciating humor (Ruch et al., 2014), and teachers characterize them as more assertive, attention-seeking, and unruly (Ruch et al., 2014). They are almost always differentiated as interfering with the classroom climate, and are regarded by most of their teachers as presenting a disciplinary problem (Cohen and Fish, 1993; Hobday-Kusch and McVittie, 2002; Ruch et al., 2014). The bestowal of the “class clown” label and the negative attributes that accompany it can be of concern, in that research has found that boys who frequently clowned in the classroom received more negative criticism from their teachers even when they weren't clowning (Yarrow et al., 1971). Students who exhibit “clowning” behavior are labeled “problematic” by their teacher because they require that a disproportionate amount of time and attention be devoted to them (Cohen and Fish, 1993; Ruch et al., 2014; Platt et al., 2016). In studies with primary grade teachers, children who teachers considered to be “aggressive,” “impudent,” “impulsive,” “lazy,” (Brophy and Good, 1974) and “non-conforming” (Helton and Oakland, 1977) were least preferred and were students who teachers most often nominated to leave their classroom. Teachers' overriding concern was that there would be a “behavioral contagion” or “ripple effect” originating from the unruly student's presence in the classroom (Safran and Safran, 1984). They will have doubts about whether these “difficult” students are able to participate and embrace the learning being offered, and will come to question whether they even belong in the classroom.

The descriptors seen as characterizing class clowns (Damico and Purkey, 1978; Ruch et al., 2014), and the behaviors that have been attributed to them, are virtually indistinguishable from those that have been empirically found to characterize the playful child. The use of humor has consistently shown to be a quality of playful children, as has their impulsivity, spontaneity, disobedience, verbosity, reactivity, and aggressiveness (Barnett, 1991b; Rogers et al., 1998). It seemed likely that the designation of “class clown” might be disproportionately attached to more playful children, and this was identified as an additional query in the study. We also considered the “class clown” label, and its association with playfulness, from the perspectives of the child, the teacher and of peers, wondering whether these perceptions might be divergent. This was also an important purpose of the study, as virtually all previous empirical studies have explored what it means to be the “class clown” from the perspective of older children and adolescents. We anticipated that if similar findings were discovered for younger children, they might stimulate longitudinal investigations as children aged developmentally.

#### Playful boys and girls in the classroom

There are a number of indications, drawn from diverse literatures, that the constructs under scrutiny in the present study might yield differential outcomes for playful boys and girls. There is a voluminous body of literature demonstrating differences between boys and girls in a multitude of variables within and surrounding the school classroom. The amount and quality of teacher-student interactions, even in kindergarten, has been shown to be different for boys and girls (Jones and Dindia, 2004), and they are generally viewed as more difficult to manage (Matthews et al., 2009), and more disruptive in the classroom (Jones and Dindia, 2004) compared to girls. From the early grades on, teachers report, and have been consistently observed, to use significant amounts of negative feedback in the classroom to try to control boys' behaviors (Jones and Dindia, 2004).

Despite the plethora of research consistently documenting sex differences in play from birth through adolescence (Hughes, 2010), the literature investigating differences between boys and girls in their magnitude, scope, or expression of playfulness is sparse. The few studies of global playfulness with preschool and kindergarten-aged children have generally not detected any sex differences (Lieberman, 1977; Barnett, 1990, 1991a). However, some sex differences have been detected among the component playfulness dimensions (Barnett, 1991b). Boys were shown to exhibit heighted physical spontaneity, while girls surpassed them in social spontaneity, however, in the three other playfulness components no differences were observed. The high prevalence of boys, and not girls, among children labeled “class clown” (Yarrow et al., 1971; Damico and Purkey, 1978; Ruch et al., 2014; Platt et al., 2016) would also portend that sex differences would be evident in assigning this label to playful children. The presence of sex differences was thus a pervasive inquiry throughout the study in each of the different outcome variables and their relationships with playfulness.

## Methods

### Participants

Participants were 278 children and their parents and teachers (*n* = 43) in kindergarten through third grades from six Midwestern public elementary schools. School records indicated that a large majority of the children were White (81%, Black = 15%, Hispanic = 1%, bi-racial = 2%, >1% of another race/ethnicity), and there were slightly more females than males (54%, *n* = 150). The age range of the children corresponded to their grade level at the time of testing (kindergarten: M = 5.7 years, SD = 0.42 years, *n* = 299; first grade: M = 6.6 years, SD = 0.61 years, *n* = 295; second grade: M = 7.6 years, SD = 0.64 years, *n* = 289; third grade: M = 8.7 years, SD = 0.70 years). Two-thirds of the children (68%, *n* = 189) were currently residing in two-parent homes at the time of testing, and 94% (*n* = 261) had at least one parent who was employed full-time. The sample, as well as individual classes and grades, had a normally distributed range in socioeconomic level, with the largest percentage (22%, *n* = 61) of family annual gross income falling within the $40,000 to $75,000 bracket (range = $15,000 to >$150,000).

A total of 43 teachers (*n*_kindergarten_ = 12, *n*_1stgrade_ = 11, *n*_2ndgrade_ = 10, *n*_3rdgrade_ = 10) provided data for the study, the vast majority of whom were female (95%, *n* = 41) and self-identified as White (93%, *n* = 40), with only a few others indicating Black (5%, *n* = 2) or bi-racial (2%, *n* = 1). There were no Hispanic/Latino teachers or Native Americans participating in the study. Teachers had been in the profession for an average of 14.90 years (range = 4–27), and in the focal school for 8.340 (range = 2–18) years. None of the teachers resided in a neighborhood below the median income level. Preliminary ANOVA tests detected no differences between teachers across or within grades on any of the demographic measures (all *p* > 0.05).

### Measures

#### Playfulness

Children were measured on their degree of playfulness utilizing the Children's Playfulness Scale (CPS; Barnett, 1990), which has been validated for children between the ages of 27 and 68 months (Barnett, 1990, 1991a; Trevlas et al., 2003). The scale consists of 23 descriptive statements to which teachers (or parents) respond utilizing a 5-point scale with responses labeled “sounds exactly like the child,” “sounds a lot like the child,” “sounds somewhat like the child,” “sounds a little like the child,” and “doesn't sound at all like the child.” Responses to the items are summed after inverted coding on designated items, such that higher scores indicate a greater degree of playfulness. Factor analyses with preschool and kindergarten-aged children have shown that the 23-item scale is comprised of the five dimensions of “physical spontaneity” (e.g., “The child is physically active during play”), “social spontaneity” (e.g., “The child plays cooperatively with other children”), “cognitive spontaneity” (e.g., “The child invents his/her own games to play”), “manifest joy” (e.g., “The child demonstrates enthusiasm during play”), and “sense of humor” (e.g., “The child enjoys joking with other children”). In the present study, the internal consistency for the total CPS score for each grade was highly satisfactory (kindergarten: 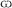 = 0.92, first grade: 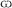 = 0.91, second grade: 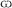 = 0.91, third grade = 0.93), as were reliability coefficients for each dimension within each grade (ranges across dimensions for kindergarten: 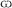 = 0.88–0.95, ranges across dimensions for first grade: 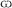 = 0.87–0.93, ranges across dimensions for second grade: 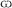 = 0.88–0.94, ranges across dimensions for third grade: 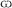 = 0.90–0.93). [The omega statistic, as a measure of internal consistency, has been found to be more appropriate and preferable to Cronbach's coefficient alpha (Huysamen, 2007; Sijtsma, 2009; Dunn et al., 2014)]. In addition, confirmatory factor analysis replicated the 5-factor structure (available from the author) of the CPS and corroborated previous findings (Barnett, 1990, 1991a,b; Trevlas et al., 2003), validating its use with the present sample.

#### Peer-rated social status

Children's social status was assessed using a peer-rating measure similar to that used by Asher et al. (1979) with children in kindergarten through third grades (Eisenberg et al., 2000). With the help of a research assistant, children rated classmates on a 4-point scale (4 = “You play with the child a lot—he or she is like a best friend” to 1 = “You do not play together because you don't want to”). Ratings by same-sex raters were averaged, as were ratings by other-sex raters, and these two scores were then averaged, following procedures utilized in previous research (Eisenberg et al., 2000). Children with parental consent were asked to rate all other children with consent, and those without consent were asked to rate their desire to play with the same number of characters from popular media familiar to their age group. A social status score for each authorized child was determined from the mean score across the peer raters. The internal consistency (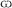) of the scale for the children with consent was 0.88 in first grade, 0.81 in second grade, and 0.84 in third grade.

#### Self-rated social competence

Children's self-perceptions about how accepted or popular they were with their peers was assessed from their scores on the six items comprising the Social Competence subscale of the Self-Perception Profile for Children (SPPC; Harter, 1982, 1985). In each item pair, one statement depicted a child who was more socially accepted, and the contrasting statement portrayed a child who was less so (e.g., “Some kids find it hard to make friends” BUT “Other kids don't find it hard to make friends”). Once the child chose a statement, he or she was then asked to indicate the extent to which the statement was like him or her (“sort of true” or “really true”). Each statement was assigned a value between “1” and “4,” with a higher score indicating more social competence. Following the recommendation of the scale's author, an assistant read each question to children at all grade levels. Reliability and validity of the scale and subscales with young children (early elementary school ages) have been well-documented (Muris et al., 2003; Harter, 2012a). The SPPC Social Competence subscale has been found to correlate significantly with ratings of children's acceptance by peers and teachers (Harter, 1985). Internal consistency reliability for the present sample was good in each grade (first grade: 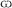 = 0.90, second grade: 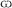 = 0.86, third grade: 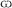 = 0.85).

#### Teacher-rated social competence

Teachers' perceptions of children's social competence were assessed by the teachers version of the SPCC scale (Harter, 1982, 1985, 2012a). The Social Competence subscale was utilized, and item (“This child finds it hard to make friends” vs. “For this child it's pretty easy to make friends”) and response (“really true” and “sort of true”; 4-point scale) formats were identical to the children's version (see above). A total mean score reflecting the teacher's perception of the child's social competence was used in the analyses. For the sample of teachers, the 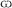 values of this subscale were 0.90 for first grade, 0.86 for second grade, and 0.87 for third grade.

#### Peer-rated classroom disruptive behavior

A measure of perceptions of their classmates' disruptive classroom behaviors was developed from the DBR-SIS (see above). Specific behaviors that comprised the disruptive behavior category were generated from previous DBR-SIS research (Riley-Tillman et al., 2009; Christ et al., 2011), and wording of the items was simplified to be appropriate for the age of the children. The eight disruptive acts presented to children were: “gets out of his/her seat without permission,” “talks or yells about things we're not working on,” “makes sounds (like humming, laughing, whistling) that aren't allowed during class time,” “talks to other kids when we're not allowed to,” “calls out things to the teacher without permission to talk,” “does or says things that interrupt what we're doing,” “is rude or mean to the teacher,” and “plays with things at his or her desk that don't have anything to do with our work.” Children were asked to rate each of their peers on each behavior using a 3-point response scale of “never,” “sometimes,” and “a lot/always.” The scale was administered to each child individually with the help of a graduate assistant and was completed over contiguous 3-day sessions. Responses to the items were summed, with a higher score indicating disruptive behaviors in the classroom were exhibited very often. The internal consistency reliability of the scale was satisfactory for all grades in the study (first grade: 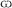 = 0.79, second grade: 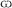 = 0.82, third grade: 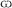 = 0.87).

#### Self-rated classroom disruptive behavior

Children rated themselves on the extent to which they felt they exhibited disruptive behaviors in their classroom utilizing the same modified DBR-SIS scale used for peer assessments (see above). At the end of the second day after providing ratings for their classmates, the assistant asked the child “What about you—how much do you think YOU do this' for each of the eight disruptive behaviors. The same 3-point response scale was used, and a total score was calculated to indicate the child's perception of how often he or she exhibited disruptive classroom behaviors. The internal consistency for these self-ratings was acceptable for all grades (first grade: 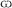 = 0.92, second grade: 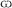 = 0.89, third grade: 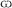 = 0.88).

#### Teacher-rated disruptive student behavior

In each grade, teachers were asked to rate students' classroom behaviors using the DBR-SIS (Direct Behavior Rating—Single Item Scales; Riley-Tillman et al., 2009; Chafouleas, 2011) following four 2-h instructional sessions toward the end of the school year. At the end of each class period teachers were asked to estimate how often each student showed disruptive behavior on a 5-point scale (1 = “never/almost never,” 3 = “sometimes,” 5 = “always/almost always”). Disruptive behavior was defined as “student action that interrupts regular school or classroom activity, such as students getting out of their seat, fidgeting, and yelling” (Johnson et al., 2016, p. 43). The DBR-SIS was selected because of its favorable reliability and validity ratings for kindergarten through eighth grade children across different raters over time, as well as its ease of use (for a review see Johnson et al., 2016). In the present study, reliability across the four rating sessions (first grade: 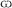 = 0.88, second grade: 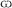 = 0.90, third grade = 0.87) was acceptable at each grade.

#### Peer rating of child as class clown

Children in each grade were seated with an assistant and read the following script: “Most classrooms have a few students who joke a lot and try to make others in the room laugh. Sometimes these students are funny and sometimes they are not really funny. Please tell me the names of students who clown around a lot of the time.” If a child received 25% or more of students' nominations in a classroom he or she received a score of “4” indicating the “class clown” designation (Fang, 2001). If a child received 15–24% of classmates' nominations a score of “3” was assigned, indicating some but not consistent regard as a class clown. For 5–14% of peer nominations, a score of “2” was given, and students receiving 4% or fewer nominations were attributed a score of “1” indicating the child was not commonly regarded as a class clown.

#### Self-rating as class clown

In addition to being asked about their classmates' clowning behaviors, the question was posed to children by an assistant: “What about you—do you think you clown around in your class a lot of the time, some of the time, or not at all?” The children's responses were scored from “1” to “3,” with higher scores indicating self-perceptions of more frequent clowning behaviors in the classroom.

#### Teacher rating of child as class clown

Teacher ratings were obtained using a different format, with classroom children's names and a 4-point Likert-scale presented. At the top, the same initial statements were provided: “Most classrooms have a few students who joke a lot and try to make others in the room laugh. Sometimes these students are funny and sometimes they are not really funny.” Teachers were then asked to rate each child utilizing the four response options of “child does this almost all of the time” (scored as “4”), “child does this a lot of the time” (scored as “3”), “child does this some of the time” (scored as “2”), and “child does this seldom or never” (scored as “1”). This rating scale was intended to be comparable to that provided by peers in that higher scores signified stronger perceptions of the child as a class clown. The mean score was utilized in statistical analyses.

#### Demographic characteristics

Demographic information was obtained from a parent questionnaire, which contained questions including the age and sex of the child, number of months of preschool they attended, and age and sex of each sibling currently in the home. Each child's birth order was determined from this information, in recognition of early research detecting such differences in play with preschool and kindergarten aged children (Moore et al., 1974).

### Procedures

#### Data collection

Data were collected as part of a larger study exploring family and sibling interrelationships in school readiness, academic skills, and social competence in public elementary schools in the midwestern United States. After obtaining approvals from universities, school district administrations, principals, kindergarten through third grade teachers, and parents, assessments for teachers and questionnaires for parents were distributed. After three follow-up mailings, a response rate of 71% from parents, and 83% from teachers was obtained. Teachers were instructed that questionnaires could be completed in their free time when they were able to concentrate, following a typical school day (provided no special “incidents” occurred either in the classroom or school), and they should only respond to questions about children whom they felt they knew well. Teacher assessments were administered toward the end of each of the academic years so that they were based on numerous interactions with each child. Teachers were compensated for their time and thanked for their participation.

Children completed instruments individually toward the end of an academic year with the help of graduate research assistants who were blind to the purposes of the study. For each questionnaire they read the instructions and items aloud, provided examples of how a child might respond, and assisted with recording responses. Data was collected over a 2-week time period. Prior to testing, graduate assistants underwent training that included viewing and discussing videotapes of children (not in the final sample) on all assessments and rehearsing questions and situations that might occur. Coders were required to achieve at least 90% for both inter- and intra- rater reliability conducted with a sampling of eight videotaped children on all measures. Children were provided with a gift card of their choice from either a local toy or book store, in consultation with their parent. Children whose parent either did not respond or who declined consent were provided with questions about their likes and dislikes in toys and play. They also received compensation following consent from a parent.

#### Missing data

From one grade to the next, some missing data occurred because a focal child moved away or left school for other reasons. To examine whether sample attrition influenced the results, three groups of children with complete data (randomly chosen n of 40), those missing data at one time period (*n* = 39), and individuals missing data at two or more times (*n* = 14) were compared (utilizing 1-way multivariate F-tests for continuous data and chi-square tests for frequency data) on all outcome measures and control (demographic) variables. None of these comparisons showed a statistically significant difference (all *p* > 0.05); it was thus concluded that there were no differences in the demographics or outcome measures due to the study procedures, and hence generalizability was not likely affected. Only children with complete data at all grade levels (*n* = 278) were included in subsequent data analyses.

### Data analysis strategy

The research design involved children and teachers who were nested in classrooms that were nested in schools. Initially, the 1,228 children who participated in the study were nested within four kindergarten classrooms within five schools, in first grade they were nested in three classrooms, in second grade they were in another three classrooms, and by grade three, these same children were in three other classrooms within the same school. No more than six children from the kindergarten class remained in the same first, second, and third grade classrooms together in any school.

Hierarchical Linear Modeling (HLM; Raudenbush and Bryk, 2002) was employed to account for the nesting of multiple observations per child, children in classrooms, and classrooms within schools. Model testing began with tests of simple unconditional models without the playfulness or sex predictors to determine intraclass correlations (ICCs) for all outcome variables. The ICC is an indication of the amount of variance that can be explained at each level, i.e., the extent to which children's outcome scores may be alike due to membership in the same classroom or school. Inspection of the ICC coefficients afforded decisions to be made about the number of levels that should be included in the final model.

Two conditional models with playfulness and sex predictors were tested, with the second adding an interaction between them. Comparisons between these models for all outcome measures indicated that for virtually all measures (the exception being first grade teachers' social competence ratings) the interaction term improved the model, as determined by it having the lowest Akaike Information Criterion value (AIC = 187.083; Vrieze, 2012), and hence the Playfulness x Sex interaction was retained. Initial conditional models included four potential covariates (experience in preschool, number of brothers and sisters, birth order) however none of these variables was a significant predictor of any of the outcome scores. Since these controls failed to reach statistical significance for all measures, they were excluded from final model construction to maintain parsimony.

The spline (or piecewise) extension of HLM (Raudenbush et al., 2011) allowed estimation of one model for each outcome at each grade and interval (thus assessing change from one grade to the next). As in typical piecewise regression frameworks, the specification allowed for separate slope estimates for each grade. To explore changes from one grade to the next we constructed the two time intervals of first to second grade (interval one), and second to third grade (interval two). The pattern of coefficients reflected in changes in each assessment during the interval of first to second grade and the interval from second to third grade were modeled as a function of the child's playfulness and/or sex.

At Levels 1 (within children) and 2 (between children), the predictor variables of playfulness (continuous) and sex (dichotomous) were person-centered and grand mean-centered, respectively (Raudenbush and Bryk, 2002). Person-centering afforded the opportunity of assessing change within the child from one grade to the next, as it reflects deviations from each child's own score. Grand mean-centering at Level 2 focused instead on how the child differed from other children on each outcome variable. Final models were tested for violations of the assumptions of HLM and none were found to deviate significantly (all *p* > 0.05).

In the event of a significant interaction, recommended procedures (Raudenbush and Bryk, 2002) involving dismantling the interaction into its component parts was followed to facilitate interpretation. Dummy coding sex (boys = 1) eliminated collinearity concerns so that significant interactions could be inspected for interpretation of main effects (Aiken and West, 1991). Procedures proposed by Hochberg (1988) were adopted, as they'd been shown to be the most appropriate for repeated measures designs with correlated outcome variables (Lix and Sajobi, 2010), and involved making an adjustment to conventional alpha levels (0.05, 0.01) in order to control for family-wise error rate. The 0.01 alpha level (Hochberg adjustment for 0.01 alpha = 0.0089) was set as the minimum for consideration of statistical significance in all HLM analyses.

## Results

### Preliminary findings

#### Descriptive statistics

Calculations of skewness and kurtosis were conducted for all measures and inspected for deviations from normality. None of the outcome measures had skew or kurtosis indices that exceeded accepted values (skew ranged from 0.09 to 1.78; kurtosis ranged from −0.23 to 1.60), suggesting no significant departures, and hence no transformations were deemed necessary (Field, 2013). Means and standard deviations for all outcome variables are shown in Table 1 by sex and grade. Correlations between the total playfulness score and all measures were calculated for boys and girls separately across grades, and are provided in Table 2. They reveal a number of distinct differences between boys and girls in their perceptions of playfulness and their classroom clowning peers. Boys perceived the playful characteristic to relate positively to sociability and to awarding of the class clown label, and not to disruptiveness in the classroom. Girls, in contrast, recognized few associations between these variables and playfulness or class clown qualities. For teachers, playfulness was strongly correlated with all of the outcome variables, however, many showed inverse relationships, in contrast to the opinions held by the children. None of these significant interrelationships presented an impediment to statistical analyses, as HLM accommodates a lack of independence between variables (Raudenbush and Bryk, 2002).

**Table 1:** Means and standard deviations for boys and girls at each grade on all outcome variables.

	**BOYS (*****n*** = **135)**						**GIRLS (*****n*** = **143)**					
	**First grade**		**Second grade**		**Third grade**		**First grade**		**Second grade**		**Third grade**	
	***M***	***SD***	***M***	***SD***	***M***	***SD***	***M***	***SD***	***M***	***SD***	***M***	***SD***
Social competence (SR)a	2.79	0.81	2.91	0.78	2.83	0.64	2.93	0.71	3.11	0.62	3.26	0.55
Social competence (TR)a	2.36	0.65	2.43	0.52	2.12	0.69	2.92	0.58	3.24	0.36	3.57	0.33
Social status (PR)^a^	2.33	0.28	2.49	0.37	2.28	0.54	2.37	0.32	2.41	0.43	2.69	0.64
Disruptive (SR)^b^	1.43	0.84	1.51	0.73	1.63	0.59	1.21	0.51	1.29	0.57	1.14	0.42
Disruptive (PR)^b^	1.18	1.09	1.22	0.97	2.13	0.66	1.03	0.53	1.17	0.79	1.21	0.51
Disruptive (TR)^c^	3.57	0.71	3.72	0.86	4.21	1.02	1.15	0.42	1.09	0.58	1.22	0.71
Class clown (SR)^b^	1.26	0.29	1.28	0.60	1.31	0.47	1.20	0.44	1.18	0.58	1.17	0.34
Class clown (PR)^a^	2.72	0.76	2.59	0.92	3.27	1.18	2.16	0.62	1.38	0.79	1.19	1.08
Class clown (TR)^a^	3.20	0.73	3.14	0.58	3.45	0.62	1.37	0.39	1.21	0.40	1.18	0.58

*SR, self-rated; TR, teacher-rated; PR, peer-rated*.

a *4-point scale*.

b *3-point scale*.

c *5-point scale*.

**Table 2:** Partial correlations between kindergarten playfulness and outcome variables for boys (upper diagonal; *n* = 135) and girls (lower diagonal; *n* = 143) across grades.

	**1**	**2**	**3**	**4**	**5**	**6**	**7**	**8**	**9**	**10**
1. Playfulness	–	013	−001	406^**^	396^**^	−295^**^	289^**^	308^**^	361^**^	402^**^
2. Disruptive (SR)	009	–	298^**^	−299^**^	256^*^	−317^**^	043	041	019	283^**^
3. Disruptive (PR)	−010	225^*^	–	−258^*^	097	−306^**^	−307^**^	046	155^*^	362^**^
4. Disruptive (TR)	018	279^**^	−236^*^	–	362^**^	−405^**^	−328^**^	−015	313^**^	340^**^
5. Social competence (SR)	107	−105	−102	008	–	−326^**^	288^**^	−014	290^**^	−296^**^
6. Social competence (TR)	−031	−287^**^	−405^**^	−279^**^	284^**^	–	−264^*^	−023	279^*^	−311^**^
7. Social status (PR)	156^*^	−104	−089	−213^*^	062	−295^**^	–	088	275^*^	−288^**^
8. Class clown (SR)	019	007	061	245^*^	027	−039	068	–	296^**^	315^**^
9. Class clown (PR)	004	095	273^**^	256^*^	091	−088	310^**^	297^**^	–	−322^**^
10. Class clown (TR)	−023	276^**^	288^**^	278^**^	046	−294^**^	335^**^	023	−288^**^	–

*Decimals omitted; covariates of experience in preschool, number of brothers and sisters, birth order controlled*.

*SR, self-rated; TR, teacher-rated; PR, peer-rated*.

** *p < 0.001*;

* *p < 0.01*.

#### Initial differences in playfulness

Kindergarten playfulness data was inspected for initial sex differences to provide some insight into whether the teachers evaluated playfulness differently in the boys and girls when they were of preschool age and began participation in this study. Results confirmed previous findings (Barnett, 1991b) in detecting no sex differences in total playfulness with children of kindergarten age [*t*_(276)_ = 1.39, *p* > 0.05].

#### Determining levels for HLM analyses

Preliminary analyses utilizing HLM were conducted to explore the influence of classroom- and school- level variability since it can be argued that children enter school varying widely in their abilities, which may differ by their classroom or school (Christian et al., 2001; Raudenbush and Bryk, 2002). The intraclass correlation coefficients (ICC; the portion of the total variance allocated to differences between schools, and between classrooms) revealed they were considered “small” (Hox, 2002), ranging from 0.001 to 0.028 for schools, and 0.002 to 0.017 for classrooms (Table 3). In general, these coefficients divulged that at most 3 and 2% of the variance occurred between classrooms and schools, respectively. Therefore, for all outcomes, the vast portion of variance was attributed to individuals within classrooms (ranging from 95 to 99%). HLM analyses for all outcome variables thus proceeded without further testing for classroom or school differences, and these levels were eliminated in subsequent models.

**Table 3:** Intra-class correlations (ICC) for individual, classroom, and school levels for all outcome measures (*N* = 278).

	**Individuals**	**Classes**	**Schools**
Social competence (SR)	0.987	0.002	0.011
Social competence (TR)	0.969	0.014	0.017
Social status (PR)	0.963	0.015	0.022
Disruptive (SR)	0.991	0.010	0.001
Disruptive (PR)	0.956	0.016	0.028
Disruptive (TR)	0.990	0.008	0.002
Class clown (SR)	0.988	0.010	0.002
Class clown (PR)	0.982	0.015	0.003
Class clown (TR)	0.974	0.017	0.019

*SR, self-rated; TR, teacher-rated; PR, peer-rated*.

### Primary findings

#### Social competence and status

Children's social competence and status were explored by examining their own assessments as well as those of their peers and teachers. The findings revealed that children's self-perceptions of their social competence were predicted by how playful they were in first and second grades, and sex was a further consideration in third grade (Table 4A). Playful boys and girls viewed themselves as more socially competent than their less playful counterparts in first and second grades, while in third grade more playful boys viewed themselves as least socially competent. As they progressed from first to second grade they didn't perceive there to be much change, however, the dramatic downturn was evident in moving from second to third grade for the playful boys.

**Table 4:** Hierarchical linear modeling results for social competence and status (*N* = 278).

	**First grade *b* (SE)**	**Second grade *b* (SE)**	**Third grade *b* (SE)**
**A. SOCIAL COMPETENCE: SELF-RATED**			
**Grade (Intercept)**			
Mean initial score	12.42^**^ (0.214)	13.79^**^ (0.117)	20.41^**^ (0.082)
Sex	0.00 (0.131)	0.01 (0.028)	0.15^*^ (0.121)
Playfulness	0.16^*^ (0.018)	0.17^*^ (0.070)	0.01 (0.098)
Playfulness x Sex	0.01 (0.112)	0.02 (0.022)	0.14^*^ (0.037)
**Change From First to Second Grade (Slope)**			
Change in average score	1.51 (0.079)		
Sex	0.00 (0.108)		
Playfulness	0.05 (0.059)		
Playfulness x Sex	0.04 (0.046)		
**Change From Second to Third Grade (Slope)**			
Mean change in average score	5.15^*^ (0.141)		
Sex	0.00 (0.158)		
Playfulness	0.02 (0.114)		
Playfulness x Sex	0.17^*^ (0.059)		
**B. SOCIAL STATUS: PEER-RATED**			
**Grade (Intercept)**			
Initial score	14.18^*^ (0.203)	13.55^*^ (0.099)	13.67^*^ (0.051)
Sex	0.01 (0.098)	0.00 (0.103)	0.14^*^ (0.108)
Playfulness	0.27^**^ (0.060)	0.34^**^ (0.051)	0.02 (0.099)
Playfulness x Sex	0.00 (0.082)	0.02 (0.087)	0.15^*^ (0.031)
**Change From First to Second Grade (Slope)**			
Mean change in average score	0.97 (0.027)		
Sex	0.02 (0.005)		
Playfulness	0.00 (0.038)		
Playfulness x Sex	0.03 (0.071)		
**Change From Second to Third Grade (Slope)**			
Mean change in average score	16.24^**^ (0.019)		
Sex	0.02 (0.081)		
Playfulness	0.03 (0.072)		
Playfulness x Sex	0.15^*^ (0.035)		
**C. SOCIAL COMPETENCE: TEACHER-RATED**			
**Grade (Intercept)**			
Initial score	22.59^**^ (0.081)	20.34^**^ (0.029)	17.38^**^ (0.067)
Sex	−0.40^**^ (0.114)	−0.09^*^ (0.078)	−0.21^**^ (0.164)
Playfulness	−0.01 (0.032)	−0.11 (0.053)	−0.02 (0.056)
Playfulness x Sex	−0.01 (0.089)	−0.38^**^ (0.042)	−0.37^**^ (0.098)
**Change From First to Second Grade (Slope)**			
Mean change in average score	13.54^*^ (0.223)		
Sex	−0.01 (0.134)		
Playfulness	−0.02 (0.040)		
Playfulness x Sex	−0.16^*^ (0.072)		
**Change From Second to Third Grade (Slope)**			
Mean change in average score	19.88^**^ (0.175)		
Sex	−0.15^*^ (0.067)		
Playfulness	−0.16^*^ (0.052)		
Playfulness x Sex	−0.22^**^ (0.059)		

** *p < 0.001*;

* *p < 0.01*.

Classmates provided another perspective on the popularity of more and less playful children. Peers were found to perceive more playful children in the first two grades as higher in social status compared to their less playful peers, with a particularly large distinction shown for boys (Table 4B). In third grade, a significant playfulness x sex interaction was found, with more playful boys viewed as lower in social status than all of their classmates. In contrast, no differences in social status as a function of the degree of playfulness were detected for girls in third grade. As children advanced from first to second grade, boys and girls who were regarded to be more playful continued to enjoy higher social status, however, this trend changed with promotion to third grade. In this latter progression, there was a decline in social status for the more playful boys, while their classmates showed an increase.

In stark contrast, teachers viewed more playful children as least socially competent in second and third grades but equivalent to their peers in first grade (Table 4C). *Post-hoc* tests for the significant interactions in the two upper grades revealed that more playful boys were judged by teachers as consistently lower in social competence compared to boys who were less playful and all girls. No such distinctions were found for playfulness in girls, and girls were consistently viewed by their teachers as more socially competent than boys in all grades. Promotion from first to second grade witnessed a significant increase in teachers' perceptions of social competence for all but the more playful boys, with a more substantial gain for all girls compared to less playful boys. For more playful boys, no change in teacher ratings of their social competence was evident from first to second grade, and they were the only children who were perceived as declining from second to third grades.

#### Disruptive classroom behavior

To address the first research question, we tested whether playfulness was related to each of the perceptions of the extent to which the child was seen as disruptive in the classroom. The analyses were conducted to examine whether the relationships differed as a function of the child's sex within and across grades (with the child characteristics of birth order, number of siblings, and preschool experience partialed out), and any changes in these scale means across grades. The HLM analyses indicated that self-rated disruptive behavior (Table 5A) was unrelated to playfulness, but boys regarded themselves as more disruptive than girls in in all three grades. Peers similarly saw no differences between more and less playful children in the first two grades, and they also thought boys showed more disruptive acts in second grade compared to girls (Table 5B). In third grade, however, they appeared attentive to the combination of playfulness and sex, in regarding playful boys as more disruptive than all other children. As children progressed from one grade to the next, this was the only relationship with playfulness noted by peers.

**Table 5:** Hierarchical linear modeling results for disruptive classroom behaviors (*N* = 278).

	**First grade *b* (SE)**	**Second grade *b* (SE)**	**Third grade *b* (SE)**
**A. DISRUPTIVE: SELF-RATED**			
**grade (intercept)**			
Initial score	1.14 (0.083)	2.06 (0.114)	1.81 (0.101)
Sex	0.21^*^ (0.026)	0.19^*^ (0.054)	0.16^*^ (0.027)
Playfulness	0.00 (0.017)	0.03 (0.066)	0.01 (0.089)
Playfulness x Sex	0.04 (0.039)	0.01 (0.109)	0.02 (0.054)
**Change From First to Second Grade (Slope)**			
Change in average score	1.76 (0.127)		
Sex	0.03 (0.058)		
Playfulness	0.02 (0.043)		
Playfulness x Sex	0.02 (0.067)		
**Change From Second to Third Grade (Slope)**			
Change in average score	1.95 (0.223)		
Sex	0.01 (0.056)		
Playfulness	0.03 (0.074)		
Playfulness x Sex	0.03 (0.022)		
**B. DISRUPTIVE: PEER-RATED**			
Initial score	1.42 (0.088)	0.99 (0.071)	1.13 (0.045)
Sex	0.01 (0.029)	0.14^*^ (0.013)	0.15^*^ (0.017)
Playfulness	0.02 (0.041)	0.03 (0.019)	0.02 (0.029)
Playfulness x Sex	0.01 (0.098)	0.10 (0.025)	0.27^*^ (0.021)
**Change From First to Second Grade (Slope)**			
Change in average score	1.84 (0.036)		
Sex	0.00 (0.107)		
Playfulness	0.01 (0.053)		
Playfulness x Sex	0.01 (0.044)		
**Change From Second to Third Grade (Slope)**			
Change in average score	13.63^*^ (0.027)		
Sex	0.06^*^ (0.119)		
Playfulness	0.01 (0.065)		
Playfulness x Sex	0.17^*^ (0.058)		
**C. DISRUPTIVE: TEACHER-RATED**			
Initial score	24.25^**^ (0.037)	24.88^**^ (0.019)	35.47^**^ (0.008)
Sex	0.37^**^ (0.065)	0.41^**^ (0.051)	0.39^*^ (0.022)
Playfulness	0.02 (0.079)	0.02 (0.068)	0.03 (0.089)
Playfulness x Sex	0.43^**^ (0.056)	0.27^**^ (0.061)	0.44^**^ (0.073)
**Change From First to Second Grade (Slope)**			
Change in average score	1.29 (0.004)		
Sex	0.23^**^ (0.089)		
Playfulness	0.01 (0.091)		
Playfulness x Sex	0.30^**^ (0.083)		
**Change From Second to Third Grade (Slope)**			
Change in average score	13.76^*^ (0.037)		
Sex	0.02 (0.034)		
Playfulness	0.00 (0.085)		
Playfulness x Sex	0.41^**^ (0.098)		

** *p < 0.001*;

* *p < 0.01*.

Teachers readily perceived differences in disruptive classroom behavior between more and less playful children, and between boys and girls, in all three grades (Table 5C). They consistently viewed less playful boys and all girls as least disruptive, and by third grade this tendency became more pronounced. As children moved from first to second grade, more playful boys were regarded as more disruptive by their teachers compared to their female counterparts and less playful others, whose assessments instead showed no significant change. When progressing to third grade, decreases in teachers' ratings of classroom disruption for almost all children were shown, the exception being playful boys whose ratings continued to increase even more sharply from second to third grades.

#### “Class clown” designation

Children appeared reluctant to assign the label of “class clown” to themselves, regardless of how playful they were or their sex. There was no distinction found between more and less playful children, or boys and girls, in children viewing themselves as the “class clown” in any grade (Table 6A). Playfulness, however, did influence peer perceptions of being a “class clown” for both boys and girls in first grade, but only for boys in second and third grades (Table 6B). After first grade, no such relationships were found between girls' playfulness and being seen as a “class clown” and there was a steep decline in girls being regarded as the class clown as children moved through the grades. In second and third grades there was an increasing tendency for boys to be viewed as a class clown compared to girls, particularly those who were more playful.

**Table 6:** Hierarchical linear modeling results for class clown ratings (*N* = 278).

	**First grade *b* (SE)**	**Second grade *b* (SE)**	**Third grade *b* (SE)**
**A. CLASS CLOWN: SELF-RATED**			
**Grade (Intercept)**			
Initial score	0.43 (0.089)	0.91 (0.021)	0.28 (0.060)
Sex	0.01 (0.044)	0.00 (0.019)	0.01 (0.022)
Playfulness	0.01 (0.080)	0.02 (0.042)	0.01 (0.058)
Playfulness x Sex	0.00 (0.074)	0.01 (0.011)	0.03 (0.082)
**Change From First TO Second Grade (Slope)**			
Change in average score	1.56 (0.104)		
Sex	0.00 (0.106)		
Playfulness	0.02 (0.088)		
Playfulness x Sex	0.00 (0.039)		
**Change From Second to Third Grade (Slope)**			
Change in average score	1.93 (0.009)		
Sex	0.02 (0.078)		
Playfulness	0.01 (0.061)		
Playfulness x Sex	0.01 (0.047)		
**B. CLASS CLOWN: PEER-RATED**			
Initial score	14.88^*^ (0.067)	16.90^*^ (0.049)	25.61^**^ (0.012)
Sex	0.15^*^ (0.077)	0.16^*^ (0.018)	0.15^*^ (0.023)
Playfulness	0.29^**^ (0.032)	0.15^*^ (0.026)	0.14^*^ (0.076)
Playfulness x Sex	0.03 (0.039)	0.15^*^ (0.019)	0.33^**^ (0.038)
**Change From First to Second Grade (Slope)**			
Change in average score	2.32 (0.034)		
Sex	0.00 (0.089)		
Playfulness	0.01 (0.071)		
Playfulness x Sex	0.00 (0.015)		
**Change From Second to Third Grade (Slope)**			
Change in average score	2.38 (0.019)		
Sex	0.03 (0.060)		
Playfulness	0.01 (0.107)		
Playfulness x Sex	0.16^*^ (0.018)		
**C. CLASS CLOWN (TEACHER-RATED)**			
Initial score	13.09^*^ (0.112)	14.22^*^ (0.065)	27.96^**^ (0.089)
Sex	0.32^**^ (0.067)	0.37^**^ (0.030)	0.36^**^ (0.049)
Playfulness	0.31^**^ (0.031)	0.32^**^ (0.076)	0.44^**^ (0.072)
Playfulness x Sex	0.28^**^ (0.069)	0.31^**^ (0.022)	0.29^**^ (0.028)
**Change From First to Second Grade (Slope)**			
Change in average score	1.18 (0.053)		
Sex	0.01 (0.103)		
Playfulness	0.04 (0.067)		
Playfulness x Sex	0.02 (0.039)		
**Change From Second to Third Grade (Slope)**			
Change in average score	1.92 (0.114)		
Sex	0.01 (0.023)		
Playfulness	0.01 (0.078)		
Playfulness x Sex	0.03 (0.045)		

** *p < 0.001*;

* *p < 0.01*.

Teacher designations of playful children as “class clown” were apparent for boys but absent for girls (Table 6C). At all grades, more playful boys were predictive of higher teacher scores as a class clown, while no relationship was found with playfulness for girls at any grade. Teachers consistently assigned the class clown moniker to boys more than girls, and to particularly playful ones compared to those who were less playful.

## Discussion and conclusions

### Summary of findings

The data are compelling in revealing that playful children are perceived by their teachers and peers very differently than their less playful classmates. In first and second grades, children who were more playful were seen by their classmates as desired playmates, inclined to be ascribed the label of class clown, but not seen as disruptive to themselves or their classroom decorum. In these same grades, the children perceived themselves to be popular among their peers, and adept in social skills. They did not see any of their playful antics as disturbances in the classroom, although they were less hesitant to assign the class clown moniker to themselves. Children did not see playful boys and girls as very different, viewing them all as more preferred play partners to their less playful peers.

In third grade, however, things took a dramatic turn. While children continued to view more and less playful children differently, they now paid careful attention to their gender, and constructed a sharp distinction between playful boys and playful girls. Most significantly, their views of boys who were very playful completely reversed, in that they now came to view them as least preferred playmates with lowest social status. And while they continued to assign them the label of class clown, peers came to view their associated clowning behaviors as disruptive activities in their classroom. In third grade, more playful girls were not any different than girls who were less playful, although the subgroup of playful boys took on their own persona, which was now predominantly negative and contrasted dramatically with how they were seen in the two prior years. The most startling (and alarming) finding was that the children themselves—most notably the playful boys—who shifted to hold increasingly negative perceptions of themselves as well by third grade. Like their peers, they came to view themselves as unpopular, and less socially skilled, compared to their classmates. Their perceptions of their classroom behaviors transformed as well, so that they now regarded them to be problematic, which had not been their perspective previously. We strongly suspect that the cause of such a substantial/considerable turnaround is rooted in the eventual influence exerted by teachers, directly and indirectly, on playful boys' self-perceptions and those of their classmates.

Beginning in first grade, teachers showed their distaste for playful boys, consistently viewing them as disruptive in the classroom and as least socially skilled, and assigning them the label of class clown. These perceptions strengthened as children progressed through their three years of school, and while most children were seen as becoming more socially competent across time, playful boys were actually regarded as declining as they approached third grade. In all grades, teachers did not view playful girls as distinct from other children—it was only playful boys that were the focus of their negative perceptions—created and continuing from the earliest school years.

### Discussion

#### Children's playfulness

One of the most significant discoveries of the study was the antipathy held by teachers for playful boys from the earliest primary grade. In all grades, teachers viewed playful boys as the most disruptive in the classroom, consistently more so than less playful boys, and all girls. At first glance, these results reinforce several streams of research conducted in school settings. In general, female teachers report a closer relationship with girls in their classroom compared to boys (Koepke and Harkins, 2008; Spilt et al., 2012) and primary school teachers have been shown to have more negative and conflictual relationships with boys in their classroom (Hamre and Pianta, 2001; Spilt et al., 2012). At all grades, boys are generally regarded by teachers as disruptive (Jones and Dindia, 2004; Esturgó-Deu and Sala-Roca, 2010; Spilt et al., 2012) and off task more frequently (Kean, 1995) than girls, which might at least partially explain their more antagonistic assessments. In addition, studies have also observed that disruptive behaviors by younger school-aged children are largely directed at teachers (Hall and Hayden, 2007), so that teachers are thus more likely to perceive playful behaviors as distracting and irritating, and in need of intercession. The finding that teachers and children differed in their views of disruptive actions in the first two grades is consistent with studies demonstrating that they often differ in their perceptions of what constitutes disruptive classroom behaviors, and the extent to which they represent a serious intrusion (Mitchell et al., 2010). A number of studies have shown that when they are aware, children don't necessarily regard disruptive classroom behaviors as undesirable or disturbing to others, and in fact might instead view them as engaging or amusing (Huesmann and Guerra, 1997). This could explain the high social status attributed to playful children by their peers in first and second grades.

The finding that it was not just boys, but rather more playful boys in particular, that incurred reactions and stigma from teachers, provides insight into the nature of playfulness for children under constricting conditions. Studies have questioned primary school teachers about the types of students they found to be problematic and those they hoped would leave school (Brophy and Good, 1974). Children who were characterized by their teachers as “aggressive,” “impulsive,” “impudent,” or “lazy,” were most frequently identified, and when asked what behaviors typify “difficult” or “problem” students, the vast majority were categorized as disruptive behaviors, with only a few related in any way to learning difficulties (Brophy and Rohrkemper, 1981). Observations in primary classrooms sought to characterize children who were most likely to extract sentiments of preference, concern, or rejection from their grade school teachers (Helton and Oakland, 1977). Results showed that teachers preferred “rigid,” “conforming,” “orderly,” “passive,” and “dependent” children, and were much more likely to reject those who were “non-conforming” or “aggressive.” Teachers are more likely to condemn behaviors directed toward themselves or other students (“aggressive,” “impudent”), particularly incessant or disruptive talking or chattering, disturbing other students, making unnecessary noise, wandering around without permission, avoiding school work, physical aggression against fellow students, and exhibiting rough or wild behavior (Safran and Safran, 1985; Hall and Hayden, 2007). The vast majority of these attributes and behaviors have been found to describe playful young children, and to uniquely distinguish them from those who are less playful (Barnett, 1991b). The qualities that have been found to be discriminating, and the constituent playfulness dimensions that emphasize the physically active, sociable, joking, impulsive, and exuberant predispositions that predominate in young playful children (Lieberman, 1977; Barnett, 1990, 1991a), appear strikingly similar to many of those found to be objectionable or intolerable by teachers. It is thus not surprising that teachers perceived more playful boys to also be more disruptive.

The perceptions of teachers that playful boys were disruptive to classroom tenor and have inferior social skills may forebode a longer-term negative trajectory for them as they move through their formal school years. Research has shown that positive student-teacher relationships relate to fewer disruptive behaviors (Wang et al., 2013), and when interactions with teachers become increasingly negative, classroom disruptions may become more frequent. Positive teacher-child relationships have also been found to be vital for children's feelings of well-being, and academic engagement and performance (Hamre and Pianta, 2006; Hughes et al., 2008). Children who experience supportive, amicable relationships with their teachers have more effectual current and future academic and social outcomes (Hamre and Pianta, 2006). Thus, playful boys who perceive negative affect or criticism by teachers may be at risk. Teachers' perceptions that playful boys have lower social competence, and a lagging rate of social development, may be communicated, and in turn impact their peer relationships (De Laet et al., 2014) and acceptance (Hughes et al., 2001; Hamre and Pianta, 2006). To the extent that this influence becomes internalized by the playful boys or their peers, a negative trajectory might be imminent with the dire longer-term outcome might be that increased problem behaviors will be observed, including delinquency and aggression (Newcomb et al., 1993) with associated feelings of loneliness, depression, and anxiety (Ladd, 2006). Thus, teachers' negative assessments of playful boys may pose ominous potential consequences for these children's social and academic development and success.

The findings in the present study that peers' assessments of playful boys were largely positive in first and second grades on all measures and then abruptly reversed to a negative course, incites disquieting concerns. These results, coupled with the observation that playful boys regarded themselves as socially skilled in first and second grades and then as socially deficient in third grade, identical to the perceptions of teachers and peers, is strongly suggestive of a social referencing transformation (Hendrickx et al., 2017) taking place. Teachers who hold and project negative attitudes toward playful boys may influence peers to embrace similar opinions by their remarks, gestures, and behaviors (Maas and Meijnen, 1999). The inverted ratings of social status, disruptive classroom behavior, and class clown branding of playful boys by peers in third grade could be construed as evidence that the classroom teacher is a dominant socializing agent who affects children's peer perceptions and relationships, particularly for those of younger age (Farmer et al., 2011). These results support and extend the literature demonstrating that students' observations of the relationships that teachers have with their classmates influences their own perceptions, affective appraisals and responses (De Laet et al., 2014; Hughes and Im, 2016).

The children's own transformed assessment of their social competence may insinuate the presence of a Pygmalion effect (Rosenthal and Jacobson, 1968) in furtherance of the literature revealing the potential that teachers have to influence children based on their beliefs about their attributes and abilities. This “invisible curriculum” (Farmer et al., 2011) focuses on the interactions between students and their teachers, and the potency of teachers' expectations for socializing students as to their behavioral conduct and performance. The essence of this proposition is that teachers form expectations for students based upon their impressions, which may or may not be accurate, and create a self-fulfilling prophesy (Jussim and Harber, 2005). While their impression might be based on a number of factors, one common and potent source is students' behavioral conduct in the classroom (Dusek and Joseph, 1983). Teachers then begin to behave differently toward certain students through the use of consistent verbal and non-verbal cues, in accord with their expectancy (Brophy and Good, 1974). Students will come to respond to this differential treatment and coordinate their behaviors accordingly, eventually internalizing their teachers' expectations (Jussim et al., 2009). This process can function directly on the student in this way, or it may be more indirect if classmates observe the distinctive feedback rendered to playful boys by the teacher. Mirroring the teacher, their classmates may treat them differently as well, and the playful boys will respond to this influence to interpret their own behavior (Brown, 2012) and to guide their future actions (Armstrong, 2011). The finding that it was not until third grade that playful boys acutely perceived and reacted to social changes in how they were viewed by their peers may be attributable to enhanced social-cognitive and socio-emotional abilities as children move through middle childhood. Studies of third graders (and older ages) have shown that children's self-understanding becomes more differentiated and they are more attuned to their social self, and receptive to social information and social comparisons with their peers (Harter, 1998, 2012b; Marsh et al., 1998). Their increasing social knowledge and the predominance assigned to peer relations have endowed them with insight into the social cues, predilections, and actions of their classmates (Rudolph et al., 1995; Harter, 2012b). Advances in perspective-taking ability would also meld so that sensitivity to peers' thoughts and feelings would be heightened with increasing age (Selman, 1980). Perhaps with these heightened abilities, peers are more likely to be aware of playful boys' behaviors and to interpret them as aberrant and/or problematic (at least in the classroom), and playful boys are simultaneously more able to process and assimilate information about peer relations.

#### Class clowns

The coincident conscription of the “class clown” label exclusively to playful boys by their teachers strongly suggests that being playful may well be maladaptive in the school classroom. The few studies that have chronicled the behaviors of class clowns have found that they are almost universally perceived by teachers as distracting and problematic, whose behaviors must be managed, shaped, or extinguished (Hobday-Kusch and McVittie, 2002; Ruch et al., 2014; Platt et al., 2016). While the findings of the study are admittedly correlational, we can posit that they are strongly indicative of a linkage between the class clown bestowal and perceptions by classroom teachers that the behaviors of playful boys are unwelcome and objectionable. The further finding that it was exclusively playful boys who were the recipient of the class clown moniker further supports the literature showing a pervasive gender disparity in ascribing this label to youth and adolescents (Fang, 2001; Platt et al., 2016). The concomitant results that playfulness in girls leads to few difficulties with teachers extends this literature to school-aged children. These data do not provide any substantive explanations as to what it is about playful boys or their specific characteristic behaviors that individualizes them from their peers—an issue that awaits empirical study. It is possible that girls respond more readily to early teacher conditioning of appropriate classroom behavior and become more adept at controlling their playful urges in comparison to boys. There is some speculative evidence from this data that this “acclimatizing” may occur to some extent in early primary grades, or to a greater extent as the child enters third grade. It is also possible that boys are more resistant to these efforts, or that their impulse or emotional control is less well developed than girls (Hines, 2004). It is also probable that teachers may shape the behavior of girls as to what is classroom-appropriate before boys at an earlier age (Sax, 2005). These may all be credible explanations for these data—rather than speculation that there are gender differences in the ability to control playful impulses.

Teachers' and peers' ascription of the “class clown” label to playful boys is worrisome in that studies have shown that the way children are labeled comes to demarcate who they are and is a strong determinant of how they feel about themselves (Becker, 1963). Labels can have a powerful effect on the behaviors and socialization of children, and if this marker is a negative one it can result in the playful boy detaching himself from his peers. If peers come to hold negative beliefs about a playful boy or about being a class clown, they may come to view the playful boy as deviating from the normative social group and exert pressure on him to either conform or conceal his playful attributes (Crocker et al., 1998). Classmates may treat the playful boy differently, or hold inaccurate expectations of him, which could lead to him experiencing social anxiety or isolation (Hirschi, 1969). In this way, the characterization of being “playful” or the designation as a “class clown” has the potential to alter the life course of a child.

### Conclusions

This longitudinal study investigated how young children's playfulness, assessed in kindergarten, was predictive of their subsequent social competence, disruptive classroom behaviors, and the designation of “class clown” from first through third grades, viewed from the lens of teachers, classmates, and themselves. The findings enlighten our understanding about playfulness in children in several ways. They extend our knowledge about playfulness to school-aged children, a developmental stage about which there is a paucity of information, and explore its predictive power with data collected over a 3-year time span. The longitudinal design of the study allowed us to reveal the fragility of playfulness in young children as the setting became increasingly rule- and adult- governed, and hence, progressively antithetical to playful expressions. Further, the initial diverging perceptions of playfulness held by teachers and peers eventually converged such that playfulness came to be regarded as deleterious to boys' social relationships and classroom behavior.

The results of this research also contribute to the “class clown” literature in a number of significant ways. The study is the first to directly link the construct of playfulness in children with the existence of the “class clown” marking in the school classroom. Several investigations have utilized one as a part of the definitional criteria for the other, yet without empirical evidence to support and explore their association. By demonstrating their synchronized appearance with the children in the study, we proffer that we have taken a first step toward this end, and in so doing hope to stimulate others to chart a sequential scientific course. In addition, to date the majority of the class clown literature has explored the application of the label to older children and adolescents, with only a few studies conducted with younger aged children. The data assessing awareness and attribution by teachers, peers, and the children themselves revealed that it was viewed in different ways and that a range from positive to negative attitudes were operative. While the “class clown” moniker was evident in all three grades, its valence changed from a positive to negative one, demonstrating its susceptibility to consequences and dissuasion. Lastly, the data reinforced the engendered nature of the class clown branding, with its increasingly exclusive application to boys in progressing from the first to third grade school years.

#### Limitations and suggestions for future research

This study is one of the first to adopt a longitudinal view of the outcomes of being playful, investigating some select social and behavioral consequences. Previous research on playfulness has identified temperament and personality characteristics of the preschool child, correlates and constitutive dimensions, and parenting styles and demographics particular to the home environment (Barnett and Kleiber, 1982, 1984; Barnett, 1990, 1991a,b; Rogers et al., 1998), yet there has been a lack of research delving into the playful predisposition in school aged children and in different types of settings. While this study has demonstrated that kindergarten playfulness is predictive of social and disruptive classroom behavior in first through third grades, caution must be exercised in adopting a causal interpretation. The implementation of an experimental design in which the playful quality could be facilitated, directed, or discouraged, and resulting social and behavioral effects detected, would enable a causal argument to be made. While the question of whether playfulness can be taught has not been resolved, much less approached, the possibility that it might be susceptible to environmental mediation is plausible. The proposition put forth that through play children's self-regulation and executive function skills are supported and enhanced (Barker et al., 2014), and that class clowns can be taught when their behavior is appropriate and when it is not (Cohen and Fish, 1993), inspires experimental research on playfulness. How playfulness can be encouraged and productively channeled in the classroom is an important question to address, as this research has begun to demonstrate the consequences of being playful for young children.

Future research should objectively chronicle the behaviors of the children in the classroom to determine whether the more playful boys were indeed acting differently than more playful girls or other boys. The negative ratings of classroom behavior for playful boys, viewed by teachers as disruptive, could be verified by objective observational assessments. The finding that peers did not initially regard the classroom behavior of playful peers in the same way as teachers, suggests that teacher expectations for classroom conduct may not have been adequately communicated to students, or that their negative perceptions were not based on tangible readily observable disobedient or mischievous actions. It would be important to determine if playfulness was viewed so negatively by teachers based on actual conduct problems or on stringent behavior expectations that differed for boys and girls. In addition, the assessment of playfulness occurred before the transition to formal schooling, so that observations of the type and extent of playful expression are essential. Evidence to suggest that playfulness has temporal or situational stability is absent, and while the data uncovered relationships with several of the outcome measures, it is crucial to be able to describe what playful behaviors are actually attempted or emitted. The ability to delineate both playful actions and disruptive behaviors in the classroom would advance this line of research considerably.

It is ardently recommended that future research on class clowns consider our procedures in response to the recommendation by Ruch et al. (2014) and Platt et al. (2016) that the construct is best defined and assessed on a continuum, rather than dichotomously as has been typical in almost all earlier research. The lack of consistency among raters in perceptions of definitional characteristics of class clowns, and/or whether a peer fits those criteria, implies that we adopt new methods and procedures. In addition to measurement issues, in the present study no consideration was given to what these divergent class clown qualities might be, which is an important avenue for further study in that some might be to be more disruptive, or asocial, or aggressive than others. For example, in their study with older children and adolescents, Ruch et al. (2014; Platt et al., 2016) identified disparate types of adolescent class clown behaviors, and clustered these into four types. Only one corresponded to generating disturbances in the classroom, and not all were rated as equally disturbing by teachers. Platt et al. (2016) speculated that class clowning behaviors could differ at different ages, and advocated for longitudinal research to explore how these behaviors might change over time. It would also be enlightening to conduct longer-term studies that follow boys identified early on as class clowns through their school years, to determine how they are able to “survive” efforts to suppress or extinguish their behaviors, and whether those who persist have suffered the ill fates we've hazarded.

The results found in the present study, as well as in others (Farnetti and Palloni, 2010), that class clowns, and perhaps more playful children, are habitually disruptive to school settings, also requires additional detailed scrutiny. While we defined what we meant by this term when the question was posed to teachers and children, it would be informative to discern whether the playful boys who were regarded as such were affected by any conditions that resulted in this perception other than a high playfulness score. For example, children with certain subtypes of ADHD (Cordier et al., 2010), problems self-regulating their behavior (McClelland et al., 2007), and those with poor inhibitory control (Ponitz et al., 2009) have also been consistently identified as disruptive and impulsive in the classroom setting. Our ability to disentangle playfulness from other comorbid conditions is paramount in continuing to hypothesize the existence of this predilection in children.

A further limitation of the study is the inadequacy of the research design to enable exploration of the role of culture, race, or ethnicity in both teacher assessments of playfulness or in classroom disruptiveness in the three primary grades. The literature is compelling in revealing cultural differences in play (Farver and Howes, 1993; Roopnarine et al., 1994; Farver et al., 1995; Farver and Shin, 1997), and in parental beliefs about children's play (cf. Lancy, 2002; Fogle and Mendez, 2006). In the current study, as in previous ones (Lieberman, 1977; Barnett, 1990, 1991b), the small number of children from any non-White ethnic group (42 Black, 3 Hispanic, 9 bi-racial) precluded any statistical testing. In addition to playfulness, there is a substantial and growing body of research about inherent biases of elementary school teachers in viewing their students' classroom behaviors. Studies have demonstrated that White teachers scrutinize Black students more than White students (Gilliam et al., 2016), they rate them as more problematic (Skiba et al., 2011; Gilliam et al., 2016) and disruptive (Thomas et al., 2008), and they impose harsher sanctions (Skiba et al., 2002, 2011; Tenenbaum and Ruck, 2007). The findings that these biases can be seen as early as preschool age (Downer et al., 2016; Gilliam et al., 2016) beseeches playfulness researchers to consider the race of the student and of the teacher in subsequent studies, and to intentionally provide for the inclusion and necessary sample sizes to investigate cultural (disentangled from social class) differences. While acknowledging the small number of Black students in this study, it remains an open question as to whether any differential perceptions of these children existed in teacher ratings of their playfulness, disruptive behaviors, or ascribing the “class clown” descriptor.

In addition to consideration of ethnicity as a salient (and potentially influential) child characteristic, there are critical teacher and classroom qualities that could also play a role and hence would be important to study. Examination of the effects of playfulness on social and behavioral outcomes did not investigate other specific types and levels of influence such as the climate of the classroom, methods of instruction, and personality of the teacher (Bierman, 2011). As there were different teachers both within and across grades, it is likely that the teacher-student relationship varied along with expectations for conduct and how explicit a hierarchy between teacher and children was in place and communicated. Hence, children's degree of classroom disruptiveness or perceptions of social competence may have varied with the characteristics of the setting (teacher, classroom, other students) such that the “antics” of more playful children, and the extent to which gender expectations were in force, may be important considerations. Children might also have been affected by the teacher's warmth or characteristic tendency to show or elicit positive affect, as has been shown to be instrumental in classroom studies (Sabol and Pianta, 2012). Future research more systematically investigating these different levels of influence, and their interaction, is needed to shed additional light on the ways in which playfulness manifests through teacher and peer perceptions and through environmental conditions, or both.

## Ethics statement

This study was carried out in accordance with the recommendations of name of guidelines, name of committee with written informed consent from all subjects. All subjects gave written informed consent in accordance with the Declaration of Helsinki. The protocol was approved by the Institutional Review Board.

## Author contributions

LB designed the study, conducted and supervised data collection and analysis, wrote the manuscript.

### Conflict of interest statement

The author declares that the research was conducted in the absence of any commercial or financial relationships that could be construed as a potential conflict of interest.
